# Validated Instruments of Quality of Life (QOL) in Patients With Acute Myeloid Leukemia (AML) and Other Cancers

**DOI:** 10.3389/fphar.2020.01109

**Published:** 2020-07-24

**Authors:** Maribel Salas, Mackenzie Henderson, Angelika Wientzek-Fleischmann, Zahidul Islam, Nora Tu, Aikaterini Bilitou, Maggie Elsharkawy, Ulf Stellmacher

**Affiliations:** ^1^ Epidemiology and Clinical Safety and Pharmacovigilance, Daiichi Sankyo, Inc., Basking Ridge, NJ, USA; ^2^ Centers for Clinical Epidemiology and Biostatistics and Center for Pharmacoepidemiology Research and Training, Perelman School of Medicine, University of Pennsylvania, Philadelphia, PA, United States; ^3^ Rutgers Institute for Pharmaceutical Industry Fellowships, Rutgers University, Piscataway, NJ, United States; ^4^ Daiichi Sankyo Europe GmbH, Munich, Germany; ^5^ HEOR/RWE: Health Economics and Outcomes Research (HEOR)/Real-World Evidence (RWE), Market Access, DSE, Munich, Germany

**Keywords:** quality of life, validation, patient-reported outcomes (PRO), review (article), instrument validation, acute myeloid leukemia

## Abstract

**Introduction:**

Acute myeloid leukemia (AML) can negatively impact quality of life (QOL). Few QOL instruments are specific to and have been validated in AML. This review aims to identify QOL instruments that have been validated in patients with AML and other cancers and summarize their psychometric properties reported in published literature. A literature review search was performed using PubMed and OVID (Biosis, Embase, MEDLINE) databases through June 25, 2020. Search terms included: QOL, health-related QOL, patient-reported outcomes *and* validity, reliability, validated, tools, instruments, test-retest, and leukemia myeloid acute, leukemia, myeloid, acute, acute myeloid leukemia. Articles were included if they focused on cancer and reported psychometric properties that could be extracted. Abstracts and their references were reviewed for inclusion.

**Results:**

Twelve evaluating ten instruments were included. Functional Assessment of Cancer Therapy Leukemia (FACT-Leu) showed internal consistency (IC) of α = 0.86 to >0.9, correlation with EQ-5D-3L of r > 0.50, correlation with European Organisation for Research and Treatment of Cancer (EORTC) QLQ-Leu of ρ = 0.29–0.63, test-retest reliability of κ = 0.861. FACT-F showed correlations with EORTC QLQ-C30 of *r* = 0.40–0.83. Hematological Malignancy Patient-Reported Outcome (HM-PRO) showed intraclass correlation coefficient (ICC) of 0.94–0.98. EORTC-8D and EQ-5D-3L showed ICC = 0.595, correlations with each other of ρ = 0.137–0.634 and with EORTC QLQ-C30 of *r* = 0.651–0.917. EORTC QLQ-C30 showed person separation reliability of 0.47 to 0.90 and patient-observer agreement of 0.85. Life Ingredient Profile (LIP) showed IC of α = 0.29–0.77 and test-retest reliability of κ = 0.42–1.0. QOL-E showed correlation with FACT-general of *R* = 0.71, internal validity of α = 0.7, and test-retest reliability of standardized Cronbach’s α = 0.7–0.92. EORTC QLQ-Leu showed IC of α = 0.6–0.79. The Acute Myeloid Leukemia–Quality of Life (AML-QOL) instrument showed IC of α = 0.72, correlations with EORTC QLQ-30 of magnitudes ρ = 0.59–0.72, and test-retest reliability of ICC = 0.52–0.91.

**Conclusion:**

Although several QOL instruments have been validated, more research is needed to determine the most clinically useful instruments in patients with AML.

## Introduction

Acute myeloid leukemia (AML) is an aggressive and devastating disease. It is estimated that only up to 27% of patients with AML in the United States survive after 5 years, and some estimates indicate a median survival for elderly patients with AML of 2 months (Menzin et al., 2002; Blum and Bloomfield, 2018). Though AML is a relatively rare cancer that accounts for about 1.2% of all cancers, it is the most prevalent type of acute leukemia in older patients in the United States (Blum and Bloomfield, 2018). In addition to the aggressive nature of the disease, patients often present with debilitating symptoms (such as fatigue, anorexia, fever, bone pain, and abnormal bleeding) that can greatly affect their ability to perform activities of daily living (ADLs) and can have a large impact on their quality of life (QOL) (Blum and Bloomfield, 2018).

AML treatment can follow one of two main pathways: intensive chemotherapy (consisting of induction and consolidation phases) or supportive care (Alibhai et al., 2007). Treatment choices are often patient-specific, especially in elderly patients for whom the benefits compared to the risks of intensive chemotherapy are largely uncertain (Alibhai et al., 2007). Furthermore, chemotherapy regimens for AML are often associated with serious treatment-related toxicities that can negatively affect QOL (Kantarjian et al., 2006). Because of this, measuring QOL in patients with AML can provide insights into aspects of their health that are adversely affected by the disease and its treatment. These measurements can help healthcare professionals make informed decisions regarding the treatment of AML.

QOL is often measured through questionnaires completed by the patients themselves (Health-related quality of life (HRQOL), 2018). Some QOL instruments are developed specifically to be used in one disease state or a group of related disease states, while others are generic instruments intended to be used in a variety of patient populations and disease states. There are few QOL instruments that have been developed for patients with hematologic disorders and fewer that have been developed specifically for patients with leukemia. However, there are many generic QOL instruments developed for general use that have been used in patients with AML in the past. Due partly to the multitude of instruments available, the appropriate QOL instruments to use in AML have not been determined. Some argue in favor of the use of generic instruments because they allow for standardization and comparability among different disease states. Others believe disease-specific instruments increase the relevance of the instrument to aspects of QOL that are differentially affected by different disease states (Lorgelly et al., 2017).

Whether using a generic or specific instrument, it is first necessary to evaluate the instrument’s validity in the population of interest to ensure that valuable information can be gained from its use. Validation of QOL instruments involves various methods to determine the extent to which the instrument measures what it purports to measure and can respond to differences in QOL over a period of change. The reliability of instruments is often measured to determine the instrument’s ability to produce stable results over a period of time during which no changes have occurred (Measurement properties: validity, reliability, and responsiveness, 2018). This literature review aims to identify QOL instruments that have been validated and summarize their psychometric properties relating to their validity and reliability in published literature.

A literature search was performed using PubMed and OVID (Biosis, Embase, and MEDLINE) to identify articles to include in this review. All databases were searched for relevant articles published through June 25, 2020. The search terms included QOL, health-related QOL, life quality, patient-reported outcomes *and* validity, reliability, validated, tools, instruments, test-retest, measurement, patient health questionnaire, questionnaire assessment *and* leukemia myeloid acute, leukemia, myeloid, acute, acute myeloid leukemia, acute myelogenous leukemia, AML. Duplicates were removed from the search results and references of included articles were reviewed to identify additional articles for inclusion. Two investigators (MS and ME) independently completed two levels of screening of articles found in the literature search. First, the titles were reviewed for relevance, and then abstracts and full texts of selected relevant articles were reviewed for inclusion. Discrepancies in the selection of abstracts were addressed through consensus. Articles were excluded if they focused on disease states other than cancer or were focused on stem cell transplants, other types of transplants, and/or other surgical procedures, or they did not report reliability and/or validity information of a QOL instrument. Articles that provided psychometric properties of validity and/or reliability in patients with cancer were included in the review.

## Results

### Article Identification and Selection

**Figure 1:**
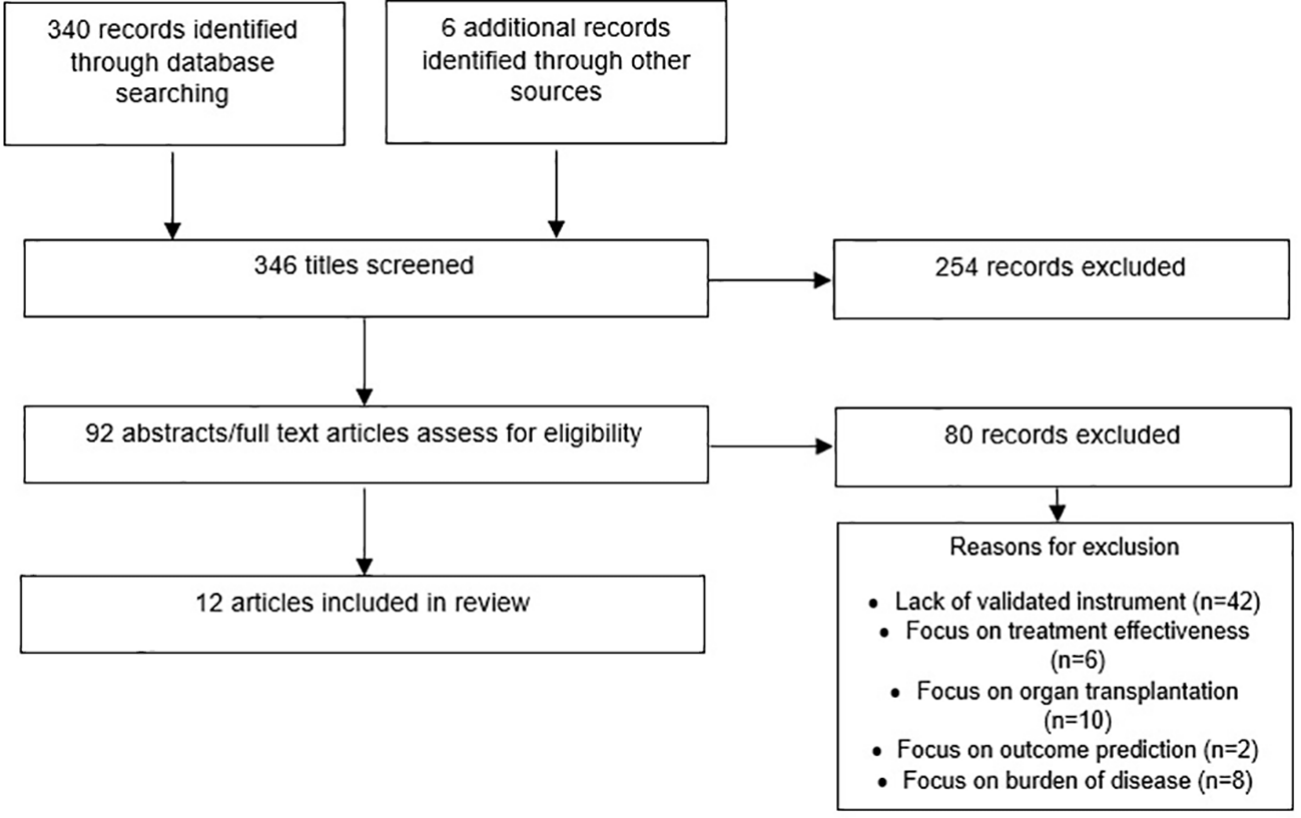
Flow of information in this review.

From a total of 346 articles found through the literature search and other sources (e.g. review of references of full articles), 92 abstracts and full texts were selected for review. Of these, 80 were excluded after abstract and full text review. The reasons for exclusion were the following: the lack of validated instrument (N = 54) or focus on: treatment effectiveness (N = 6), organ transplantation (N = 10), predictors of outcomes (N = 2), and burden of disease (N = 8). The total number of full text articles included in this review is 12 (**Figure 1**). The article selection process is documented in **Figure 1**, and the properties of the included articles are described in **Table 1**.

**Table 1:** Description of included articles.

References	Year	Design	Country	Instrument	Patient population	N
Cella et al. (2012)	2012	Prospective	United States	FACT-Leu	AML	79
Peipert et al. (2020)	2020	Retrospective review of phase 2/3 clinical trial	Europe, Australia, United States, South Korea, Taiwan	FACT-Leu	AML	317
Alibhai et al. (2007)	2007	Prospective	Canada	FACT-F	AML	65
Goswami et al. (2019)	2019	Prospective	United Kingdom	HM-PRO	Hematologic malignancy	193
Lorgelly et al. (2017)	2017	Prospective	Australia	EORTC-8D; EQ-5D-3L	Cancer	1678
Shih et al. (2013)	2013	Prospective	Taiwan	EORTC QLQ-C30	Cervical, breast, lung, liver, or colorectal cancer	2295
Groenvold et al. (1997)	1997	Cross-sectional	Europe	EORTC QLQ-C30	Gynecologic or breast cancer	95
Aaronson et al. (1993)	1993	Prospective	Western Europe, North America, Australia, Japan	EORTC QLQ-C30	Lung cancer	305
Stalfelt and Wadman (1993)	1993	Prospective	Sweden	LIP	Hematologic malignancy	35
Oliva et al. (2013)	2013	Cross-sectional	United States	QOL-E	Myelodysplastic syndromes	52 (phase 2); 147 (phase 3)
Watson et al. (1996)	1996	Cross-sectional	United States	MRC/EORTC QLQ-Leu	AML	388
Buckley et al. (2020)	2020	Prospective	United States	AML-QOL	AML and aggressive myeloid neoplasms	202 (factor analysis), 50 (validation)

AML, acute myeloid leukemia; AML-QOL, Acute Myeloid Leukemia-Quality of Life; EORTC-8D, European Organisation for Research and Treatment of Cancer Quality of Life Questionnaire 8-Dimension; EORTC QLQ-C30, EORTC Quality of Life Questionnaire Core 30; EQ-5D-3L, EuroQOL EQ-5D-3L questionnaire; FACT-Leu, Functional Assessment of Cancer Therapy Leukemia; FACT-F, FACT Fatigue Subscale; HM-PRO, Hematological Malignancy-Patient-Reported Outcome; LIP, Life Ingredient Profile; MRC/EORTC QLQ-LEU, Medical Research Council/EORTC QLQ Leukemia; QOL-E, Health-Related Quality of Life Questionnaire.

### Validated Instruments

There were ten QOL instruments with reported validity and/or reliability properties: Acute Myeloid Leukemia – Quality of Life (AML-QOL), EuroQol EQ-5D-3L, Life Ingredient Profile (LIP), Hematological Malignancy-Patient-Reported Outcome (HM-PRO), Health-Related Quality of Life (QOL-E), European Organisation for Research and Treatment of Cancer Quality of Life Questionnaire Core 30 (EORTC QLQ-C30), EORTC 8-dimension (EORTC-8D), Medical Research Council (MRC)/EORTC QLQ-LEU, and Functional Assessment of Cancer Therapy fatigue subscale (FACT-F) and leukemia (FACT-Leu). Of these, one is a generic QOL instrument, three are specific to hematologic disorders, three are specific to cancer, and three are specific to leukemia. A brief description of the specificity of the QOL instruments and the disease states the instruments were evaluated in are provided in **Table 2**.

**Table 2:** Comparison of instrument specificity and disease states in which the instruments were evaluated.

Specificity of instrument	Instrument(s)	Disease state evaluated
Generic	EQ-5D-3L	Any cancer
Specific to hematologic disorders (including malignancy)	LIP	Hematologic malignancy
	HM-PRO	
	QOL-E	Myelodysplastic syndromes
Specific to cancer	EORTC QLQ-C30	Lung, gynecologic, breast, liver, or colorectal cancer
	EORTC-8D	Any cancer
	FACT-F	AML
Specific to leukemia	FACT-Leu	
	MRC/EORTC QLQ-LEU AML-QOL	

AML-QOL, Acute Myeloid Leukemia-Quality of Life; EORTC-8D, European Organisation for Research and Treatment of Cancer Quality of Life Questionnaire 8-Dimension; EORTC QLQ-C30, EORTC Quality of Life Questionnaire Core 30; EQ-5D-3L, EuroQOL EQ-5D-3L questionnaire; FACT-F, Functional Assessment of Cancer Therapy Fatigue Subscale; FACT-Leu, FACT Leukemia; HM-PRO, Hematological Malignancy-Patient-Reported Outcome; LIP, Life Ingredient Profile; MRC/EORTC QLQ-LEU, Medical Research Council/EORTC QLQ Leukemia; QOL-E, Health-Related Quality of Life.

### General Overview of Validity and Reliability Measures

There was variation in methods used to measure validity and reliability. Measures to assess validity included convergent validity and agreement between trained observers and patients. Convergent validity measures the correlation between two instruments that are meant to measure the same construct. Agreement measures the correlation between an observer’s response to items in an instrument and the patient’s responses to items in the instrument. Measures to assess reliability included internal consistency, test-retest reliability, and intraclass correlation coefficient. Internal consistency measures the correlation of items for the same construct within an instrument. Test-retest reliability measures the correlation of a patient’s responses to items in an instrument at two different time points during which nothing significant should have changed to alter the patient’s responses. Intraclass correlation coefficient measures the correlation between items or responses within a group. Psychometric properties of validity and reliability for the instruments evaluated are presented in **Table 3** and discussed further in the next sections.

**Table 3:** Psychometric properties of the instruments reported in published literature.

Instrument	References	Measure	Validity	Reliability
FACT-Leu	Cella et al. (2012)	Internal consistency		α = 0.86–0.88
		Correlation with POMS total score	ρ = 0.74	
		Correlation with EORTC QLQ-Leu subscales	ρ = 0.36–0.60	
		Test-retest reliability		ICC = 0.861
	Peipert et al. (2020)	Internal consistency (all scales)		α≥0.70
		Internal consistency (FACT-Leu total, FACT-Leu TOI, FACT-G)		α≥0.90
		Correlation with EQ-5D-5L (except SWB)	r > 0.50	
		Correlation between FACT-Leu change scores and EQ-5D scale change scores	r > 0.50	
FACT-F	Alibhai et al. (2007)	Correlation with EORTC QLQ-C30 global QOL, physical, role, emotional, social, and cognitive function	*r*_t0_ = 0.40–0.73 *r*_t1_ = 0.54–0.83 *r*_t2_ = 0.50–0.75 *r*_t3_ = 0.50–0.77	
HM-PRO	Goswami et al. (2019)	Intraclass correlation		ICC = 0.94–0.98
		Correlation for all domains	ρ > 0.8	
EORTC-8D; EQ-5D-3L	Lorgelly et al. (2017)	Correlations between EORTC-8D and EQ-5D-3L	ρ = 0.137–0.634	
		Correlations between baseline health state values for EORTC-8D, EQ-5D-3L, and EORTC-QLQ-C30	*r* = 0.651–0.917	
		Agreement between EORTC-8D and EQ-5D-3L	ICC = 0.595	
EORTC QLQ-C30	Shih et al. (2013)	Reliability (unidimensional PCM)		PSR = 0.47–0.891
		Reliability of physical function, fatigue, global health, QOL		PSR > 0.8
		Reliability of cognitive function, nausea, vomiting		PSR < 0.5
		Reliability (multidimensional PCM)		PSR = 0.66–0.90
		Raw score correlations between EORTC QLQ-C30 subscales	*r* = 0.29–0.75	
	Groenvold et al. (1997)	Agreement between patients and observers^a^		0.85^a^
		Agreement coefficient between patients and observers^b^		κ = 0.85
	Aaronson et al. (1993)	Inter-scale correlations for physical and role functioning, fatigue	*r* = 0.54–0.63	
		Inter-scale correlations for fatigue, emotional, and social functioning	*r* > 0.40	
		Reliability in emotional functioning, global QOL, fatigue, pain (before and during treatment)		α = 0.73–0.89
		Reliability in physical functioning, role functioning, cognitive functioning, social functioning, nausea/vomiting		α = 0.54–0.77
LIP	Stalfelt and Wadman (1993)	Internal consistency (LIP 2)		α = 0.72–0.77
		Internal consistency (LIP 3)		α = 0.29–0.68
		Test-retest reliability (LIP 2 and LIP 3)		κ = 0.42–1.0
		Correlation between LIP 2, KPS, and Vitagram^c^	*r*≥0.7	
QOL-E	Oliva et al. (2013)	Correlation coefficient with FACT-G for physical, emotional, and functional well-being, overall, and treatment outcome	*R* = 0.71	
		Test-retest reliability (test, retest): Physical domain Functional domain Social domain Sexual domain Fatigue domain MDS-specific domain		SCA = 0.83, 0.75 0.80, 0.73 0.77, 0.67 0.88, 0.92 0.73, 0.75 0.78, 0.76
		Intraclass correlation		ICC = 0.65–0.80
		Internal consistency (phase 3)		SCA ≥0.70
EORTC QLQ-LEU	Watson et al. (1996)	Internal consistency for factor 1^d^		α = 0.79
		Internal consistency for factor 2^d^		α = 0.71
		Internal consistency for factor 3^d^		α = 0.60
AML-QOL	Buckley et al. (2020)	Internal consistency (physical, social, cognitive, anxiety, and depression domains)		α = 0.72–0.86
		Correlation between AML-QOL Physical and EORTC QLQ-C30 Physical Function domains at T1	ρ = 0.63	
		Correlation between AML-QOL Physical and EORTC QLQ-C30 Fatigue domain at T1	ρ = −0.62	
		Correlation between AML-QOL Cognitive, Anxiety, and Depression domains and related EORTC QLQ-C30 domains at T1	ρ = 0.59–0.72	
		Correlations between AML-QOL single item nausea and sleep scores and related EORTC QLQ-C30 domains at T1	ρ= −0.60 to −0.61	
		Test-retest reliability (all domains)		ICC = 0.52–0.91

ICC, intraclass correlation coefficient; KPS, Karnofsky performance status; ρ, Spearman’s rank correlation coefficient; PCM, partial credit model; POMS, Profile of Mood States; PSR, person separation reliability; r, Pearson product-moment correlation coefficient; SCA, standardized Cronbach’s α; SWB, social well-being; T1, timepoint 1; TOI, Trial Outcomes Index

^a^Agreement = number of times patient and observer chose same response category divided by number of times item was answered by both patient and observer; ^b^Coefficient of agreement corrected for change agreement; ^c^Carlens Vitagram index performance scale; ^d^Factor 1 = signs/symptoms of graft versus host disease; factor 2 = signs/symptoms of infection; factor 3 = sensory loss, functional status.

The Consensus-Based Standards for the Selection of Health Status Measurement Instruments (COSMIN) checklist criteria were applied to all articles to evaluate the methodological quality of the studies (Mokkink et al., 2010). These results are presented in **Table 4**.

**Table 4:** COSMIN Checklist Scores evaluating the methodological quality of each study.

Reference	Reliability			Validity			Responsiveness
	Internal Consistency	Reliability	Measurement Error	Content Validity	Construct Validity	Criterion Validity	
Cella et al. (2012)	Fair/Good	Good	–	–	Good	–	Poor
Alibhai et al. (2007)	–	–	–	–	Good	–	Fair
Goswami et al. (2019)	–	Good	–	–	Poor	–	–
Lorgelly et al. (2017)	–	–	–	–	Good	–	–
Shih et al. (2013)	–	Good	–	–	Poor/Fair	–	–
Groenvold et al. (1997)	–	Good	–	–	–	–	–
Aaronson et al. (1993)	Fair/Good	–	–	–	Fair	–	Good
Stalfelt and Wadman (1993)	Good	Good	–	–	Fair/Good	–	Poor
Oliva et al. (2013)	Fair/Good	Good	–	–	Fair	–	–
Watson et al. (1996)	Good	–	–	–	–	–	–
Peipert et al. (2020)	Good	–	–	–	Good	–	Fair
Buckley et al. (2020)	Good	–	–	Good	Good	–	Fair

Due to the variability in the methods used across studies to evaluate QOL instruments, it was not feasible to directly compare the psychometric properties of all instruments included in the review.

In addition to quantitative results, many of the articles also qualitatively evaluated characteristics of the QOL instruments. These qualitative results are reported in the following sections when applicable.

### Descriptions of Included Studies

#### AML-QOL

Buckley and colleagues (2020) developed and validated the AML-QOL, an AML-specific QOL instrument. They did this in several cohorts of patients: cohorts of three to six patients with AML or aggressive myeloid neoplasms (development), 202 patients with AML or high risk myeloid neoplasms (to evaluate internal consistency), and 50 patients with AML or high risk myeloid neoplasms (to evaluate test-retest reliability, convergent/divergent validity, and sensitivity). Patients were evaluated at five points in time: prior to treatment (T1), days 8 to 18 after treatment initiation (T2), 1 to 4 days after T2 (T3), at the end of the cycle (T4), and after all planned chemotherapy (T5). Reliability and validity results are reported in **Table 3**. During the development phase, patients and healthcare providers reported the instrument was “comprehensible, pertinent, and thorough.” Eleven items (out of 38) were removed after factor analysis with 202 patients. Furthermore, a higher/worse Eastern Cooperative Oncology Group (ECOG) performance status was reported to be associated with significantly lower summary scores on AML-QOL. AML-QOL was reportedly sensitive to change; patients who perceived a benefit showed higher QOL scores while those who perceived a worsening had lower QOL scores.

#### FACT-Leu and FACT-F

Cella and colleagues (2012) evaluated FACT-Leu, a QOL instrument developed from the FACT-General instrument. The authors evaluated FACT-Leu’s face, content, and convergent validity and reliability in 79 patients with leukemia at three points in time (baseline, 3–7 days later, 8–12 weeks later). Reliability and select validity results are reported in **Table 3**. Additionally, patients reported that the FACT-Leu was “relevant,” “comprehensive,” and “easy to understand.”

Peipert and colleagues (2020) evaluated the validity, internal consistency, and responsiveness to change of the FACT-Leu instrument in 317 clinical trial participants with AML. Validity and reliability results are reported in **Table 3**. Additionally, all but two scales on FACT-Leu were reported to decrease as ECOG performance status increased/worsened, and all but two scales on FACT-Leu were able to distinguish between baseline ECOG performance status groups. The FACT-Leu was reported to exhibit responsiveness to change; as shown by correlations between change scores for FACT-Leu and EQ-5D scales.

Alibhai and colleagues (2007) evaluated FACT-F, the fatigue subscale of the FACT-General instrument. The authors evaluated FACT-F compared to EORTC QLQ-C30, the Geriatric Depression Scale, the Edmonton Symptom Assessment Scale, and the Barthel and Lawton-Brody scales for ADLs in 65 patients with AML who were at least 60 years old. Patients were followed for up to 6 months. Correlations with EORTC QLQ-C30 subscales are presented in **Table 3**. The authors reported that fatigue scores improved slightly over time in patients receiving or not receiving intensive chemotherapy and that patients who died or withdrew from the study tended to have worse fatigue scores than those who remained in the study. Fatigue was found to be inversely correlated to global health, major domains of QOL, and ADLs. Because of the relatively short follow-up, the authors indicated that it was impossible to differentiate fatigue and QOL results due to intensive chemotherapy or the cancer itself.

#### HM-PRO

Goswami and colleagues (2019) evaluated electronic and paper versions of the HM-PRO instrument in 193 patients with hematologic malignancies (29 with AML specifically) in the United Kingdom. Patients completed the paper and electronic versions of the instrument on the same day. Correlations for the electronic and paper instruments are presented in **Table 3**. Patients reported that HM-PRO was easily readable, they could answer questions spontaneously, and they thought the electronic version of HM-PRO was easy to follow. The authors additionally reported that it took patients a median of 5 min to complete the paper version and 6.5 min to complete the electronic version of the HM-PRO.

#### EORTC-8D and EQ-5D-3L

Lorgelly and colleagues (2017) evaluated the EORTC-8D and EQ-5D-3L in 1678 patients with a variety of cancer diagnoses who were enrolled in Cancer 2015. Correlation results between the instruments and EORTC QLQ-C30 are presented in **Table 3**. Both EQ-5D-3L and EORTC-8D were reported to be sensitive to the following: sex, admission to hospital, smoking, stage of disease, hospital insurance, expected future follow-up, and Eastern Cooperative Oncology Group performance status score.

#### EORTC QLQ-C30

Shih and colleagues (2013) evaluated the reliability and construct validity of the EORTC QLQ-C30 with unidimensional and multidimensional Rasch partial credit models in 2295 patients with lung, breast, cervical, liver, or colorectal cancer. Reliability results are presented in **Table 3**. The authors reported that reliability was higher with a multidimensional partial credit model compared to a unidimensional partial credit model. Additionally, the authors reported a significant difference in deviance between the two models (*P* < 0.001), a method they used to evaluate the construct validity of the instrument.

Groenvold and colleagues (1997) evaluated the patient-trained observer agreement for the EORTC QLQ-C30 questionnaire in 95 patients with gynecologic or breast cancer. Agreement results are presented in **Table 3**. Disagreements between patients and observers were reported to fall into the following categories: observer found patient’s response surprising, patient reported symptoms were due to something else, patient did not understand how to interpret an item or the patient misunderstood an item, or the observer did not know how to respond.

Aaronson and colleagues (1993) evaluated the reliability and validity of the EORTC QLQ-C30 questionnaire in nine languages in 305 patients with nonresectable lung cancer who were considered candidates for intensive chemotherapy or radiation. Patients were evaluated at two time points, once after diagnosis before starting treatment and once during treatment. Reliability results are presented in **Table 3**. Validity was evaluated through correlations between subscales, ability to discriminate between subgroups of patients, and responsiveness to change in health status over time. The instrument did not find differences between pretreatment and on-treatment scores depending on disease stage except for emotional functioning in patients with metastatic disease compared to non-metastatic disease. Repeated-measures analysis of variance (ANOVA) did not find any differences in pretreatment or on-treatment scores on any scale except nausea and vomiting, which showed mean scores of 6 and 20, respectively (p < 0.001). However, when patients were sub-grouped by performance status, repeated-measures ANOVAs found between-group differences over time in physical functioning (*P*<.001), role functioning (*P*<.001), fatigue (*P*<.01), nausea/vomiting (*P*<.05), and global QOL (*P*<.01). About 10% of patients reported that they found at least one item on the questionnaire to be confusing or difficult to answer.

#### LIP

Stalfelt and Wadman (1993) evaluated the validity of the LIP instrument (comprising LIP 1, LIP 2, and LIP 3) in 35 patients with hematologic malignancies. Reliability and validity results are presented in **Table 3**. Patients with AML showed lower LIP 2 scores in all areas except side effects and disease symptoms during the induction phase of chemotherapy. Scores on LIP 2 mobility and autonomy items, physical symptoms, mental symptoms, and disease symptoms were reported to be significantly lower in the patients with *advanced* myeloma compared to the myeloma patient group as a whole, but no quantitative analysis results were provided. Correlations between LIP 3 and LIP 1 and 2 were low, which the authors used to convey divergent validity, as these instruments are meant to measure different aspects of life. No quantitative results were presented other than those included in **Table 3**. During the study, few items on the LIP questionnaires were reported to require reformulation due to confusion or low response rates, including those about “social contact,” “sexuality,” and “degree of energy.”

#### QOL-E

Oliva and colleagues (2013) developed and evaluated the validity of the QOL-E in three phases: a development phase, a pilot study phase with 52 patients with myelodysplastic syndromes (MDS), and a third phase evaluating the validity of the instrument in 147 patients with MDS. Validity and reliability results from the second and third phases of the study are presented in **Table 3**. The development phase identified nine concepts important to patients with MDS that were included in the instrument. During the second phase of the study, 11 items were removed because they did not fit the analysis, or they were incomprehensible or misunderstood by patients.

#### MRC/EORTC QLQ-LEU

Watson and colleagues (1996) evaluated the validity and reliability of the MRC/EORTC QLQ-LEU in 388 patients with leukemia in long-term complete remission. Patients received, completed, and mailed back one questionnaire each for the study. Reliability results are presented in **Table 3**. After analysis, the researchers recommended alteration of the item subscales in the instrument due to questionable validity.

## Discussion

A total of 12 articles (Aaronson et al., 1993; Stalfelt and Wadman, 1993; Watson et al., 1996; Groenvold et al., 1997; Alibhai et al., 2007; Cella et al., 2012; Oliva et al., 2013; Shih et al., 2013; Lorgelly et al., 2017; Goswami et al., 2019) presenting psychometric properties of validity and/or reliability for ten QOL instruments are presented in this review. Of these, eight articles evaluated seven instruments (AML-QOL, FACT-F, FACT-Leu, EORTC QLQ-Leu, LIP, HM-PRO, and QOL-E) in patients with AML or disease states related to and inclusive of AML. Of the remaining articles, one evaluated two instruments (EQ-5D-3L and EORTC-8D) in patients with any cancer diagnosis, with no indication of their validity in the AML subpopulation. The remaining three articles evaluated the EORTC QLQ-C30 in several patient populations (including those with lung, cervical, breast, liver, and colorectal cancer) but lack evidence in AML.

Articles that evaluated instruments in more specific populations tended to have smaller sample sizes, with a median of 113 patients (range, 35-388) (Stalfelt and Wadman, 1993; Watson et al., 1996; Alibhai et al., 2007; Cella et al., 2012; Oliva et al., 2013; Goswami et al., 2019; Buckley et al., 2020; Peipert et al., 2020). Articles that evaluated instruments in broader patient populations tended to have larger sample sizes, with a median of 991.5 patients (range, 95–2,295) (Aaronson et al., 1993; Groenvold et al., 1997; Shih et al., 2013; Lorgelly et al., 2017). This indicates a trade-off between evaluating instruments in the disease of interest versus evaluating instruments in a large patient sample. This limitation in published literature and lack of replicated results available restricts the ability to evaluate the true validity and appropriateness of the instruments in patients with AML. In determining what QOL instrument to use in patients with AML, the need for more robust validity information must be weighed against the need for validity information in the specific population of interest.

Validity and reliability results varied both within a single instrument and between different instruments included in this review. In general, correlation coefficients in the range of 0.3 to 0.5 can be considered to be low, those in the range of 0.5 to 0.7 can be considered to be moderate, and those in the range of 0.7 to 1.0 can be considered to be high to very high (Mukaka, 2012). Many instruments included in this review showed at least moderate values for validity and reliability outcomes. Exceptions to this were largely due to results with wide ranges that encompassed low to moderate or high values. These included FACT-Leu’s correlation with EORTC QLQ-Leu subscales (range of ρ = 0.36–0.6), correlations between EORTC-8D and EQ-5D-3L (range of ρ = 0.137–0.634), test-retest reliability for LIP 2 and LIP 3 (range of κ = 0.42–1.0), baseline FACT-F correlations with EORTC QLQ-C30 global QOL, physical, role, emotional, social, and cognitive function (range of *r*_t0_ = 0.4–0.73), and EORTC QLQ-C30 reliability (unidimensional partial credit model; person separation reliability range of PSR = 0.47–0.891).

QOL instruments can be validated in many ways, some of which were not adequately addressed in this review. One important example of this is sensitivity to change. In other words, the ability of the instrument to detect changes in QOL depending on the disease course and progression of the disease at different time points in the patient’s life (e.g., QOL in remission vs. QOL in relapse). Only two instruments were quantitatively evaluated for sensitivity to change: EORTC QLQ-C30 and FACT-Leu. EORTC QLQ-C30 was quantitatively evaluated for sensitivity to change with variable results (Stalfelt and Wadman, 1993). Changes in scores for FACT-Leu were correlated to changes in scores for EQ-5D scales, and resulted in moderate correlations (Peipert et al., 2020). Three articles offered qualitative evidence of sensitivity to change for EORTC-8D, EQ-5D-3L, FACT-F, and LIP [6,9,14]. However, sensitivity to change has been qualitatively evaluated further in patients with AML for two instruments discussed in this review, EORTC QLQ-C30 and HM-PRO. These articles were not specifically included in the review because no psychometric properties were reported. In one article, patients who later reported a QOL similar to their baseline QOL did not show changes in HM-PRO scores. However, patients who later reported a QOL that was better than their baseline QOL showed significant improvement on HM-PRO scores (Schumacher et al., 1998). In two other articles, patients’ EORTC QLQ-C30 scores were found to be related to treatment, phase of the disease, and performance status. However, due to a lack of detailed information regarding sensitivity to change, the importance of this type of validity remains unclear (Alibhai et al., 2007; Goswami et al., 2018).

Accurately measuring QOL in patients with AML can assist healthcare professionals in understanding the overall health state of their patients, which can help tailor health care to individual patient needs. Before QOL instruments can be used effectively, they must be developed and validated to ensure that they accurately measure what they intend to measure and that they can provide useful information about the patient population of interest. This review aimed to understand which QOL instruments have been used and validated in patients with AML or other types of cancer. Although many QOL instruments have been validated, the inconsistency in methods and nature of the research make it infeasible to directly compare these instruments to one another to determine which instruments are appropriate to use in patients with AML.

## Conclusion

Many QOL instruments, both generic and specific, have been validated in patients with AML or other cancers. However, certain types of validity information are still lacking for many QOL instruments, and the instruments cannot be directly compared to each other. There is a significant gap in literature evaluating the validity and reliability of QOL instruments in patients with AML. This is especially true concerning the instruments’ sensitivity to change in a patient’s clinical status and disease status. Furthermore, while more recent research has evaluated QOL instruments specifically in patients with AML, their small sample sizes and lack of replicated results makes it difficult to appropriately interpret their findings. More research is required to determine the most responsive and clinically useful instrument for patients with AML, especially in patients who relapse or are refractory or respond to treatment.

## Author Contributions

MS and ME designed and performed the literature search and evaluated articles for inclusion. MS and MH drafted the manuscript and critically reviewed the final manuscript. AW-F, ZI, NT, AB, and US discussed the draft and critically reviewed the final manuscript.

## Funding

This work was supported by Daiichi Sankyo, Incorporated.

## Conflict of Interest

The authors declare that the research was conducted in the absence of any commercial or financial relationships that could be construed as a potential conflict of interest.

The handling editor declared a past co-authorship with one of the authors MS.

The authors declare that this study received funding from Daiichi Sankyo, Inc. The funder had the following involvement with the study: the design and conduct of the manuscript was planned and performed by employees of Daiichi Sankyo, and the manuscript was written and reviewed by the authors, who were all contractors (MH) or employees (all others) of Daiichi Sankyo, Inc. or Daiichi Sankyo, GmbH during their involvement in the manuscript. ME is currently an employee of Astellas Pharma US; Astellas Pharma US did not have any involvement in this study.

## Supplementary Material

The Supplementary Material for this article can be found online at: https://www.frontiersin.org/articles/10.3389/fphar.2020.01109/full#supplementary-material

## References

[B1] Aaronson N. K. Ahmedzai S. Bergman B. Bullinger M. Cull A. Duez N. J. (1993). The European Organization for Research and Treatment of Cancer QLQ-C30: a quality-of-life instrument for use in international clinical trials in oncology. J. Natl. Cancer Inst. 85, 365–376. 10.1093/jnci/85.5.365 8433390

[B2] Alibhai S. M. H. Leach M. Kermalli H. Gupta V. Kowgier M. E. Tomlinson G. A. (2007). The impact of acute myeloid leukemia and its treatment on quality of life and functional status in older adults. Crit. Rev. Oncol. Hematol. 64, 19–30. 10.1016/j.critrevonc.2007.07.003 17765568

[B3] Alibhai S. M. H. Leach M. Kowgier M. E. Tomlinson J. M. Brandwein J. M. Minden M. D. (2007). Fatigue in older adults with acute myeloid leukemia: predictors and associations with quality of life and functional status. Leukemia 21, 845–848. 10.1038/sj.leu.2404576 17287855

[B4] Blum W. Bloomfield C. D. (2018). “Acute myeloid leukemia,” in Harrison"s Principles of Internal Medicine, 20th ed. Eds. Jameson J. AS F. DL K. SL H. DL L. Loscalzo J. (New York: McGraw-Hill).

[B5] Buckley S. A. Halpern A. B. Othus M. Walter R. B. Lee S. J. (2020). Development and validation of the AML-QOL: a quality of life instrument for patients with acute myeloid leukemia. Leuk. Lymphoma 61, 1158–1167. 10.1080/10428194.2019.1709838 31909637

[B6] Cella D. Jensen S. E. Webster K. Hongyan D. Lai J.-S. Rosen S. (2012). Measuring health-related quality of life in leukemia: the Functional Assessment of Cancer Therapy–Leukemia (FACT-Leu) questionnaire. Value Health 15, 1051–1058. 10.1016/j.jval.2012.08.2210 23244807

[B7] Goswami P. Oliva E. N. Ionova T. Salek S. (2018). Responsiveness and the minimally clinically important difference for HM-PRO in patients with hematological malignancies. Blood 132 (suppl 1), 2294. 10.1182/blood-2018-99-117094

[B8] Goswami P. Oliva E. N. Ionova T. Else R. Kell J. Fielding A. K. (2019). Paper and electronic versions of HM-PRO, a novel patient-reported outcome measure for hematology: an equivalence study. J. Comp. Eff. Res. 8, 523–533. 10.2217/cer-2018-0108 31037971

[B9] Groenvold M. Klee M. C. Sprangers M. A. G. Aaronson N. K. (1997). Validation of the EORTC QLQ-C30 quality of life questionnaire through combined qualitative and quantitative assessment of patient-observer agreement. J. Clin. Epidemiol. 50, 441–450. 10.1016/S0895-4356(96)00428-3 9179103

[B10] Health-related quality of life (HRQOL) (2018). Centers for Disease Control and Prevention. Available at: https://www.cdc.gov/hrqol/index.htm (Accessed 10 September 2019).

[B11] Kantarjian H. O’Brien S. Cortes J. Giles F. Faderl S. Jabbour E. (2006). Results of intensive chemotherapy in 998 patients age 65 years or older with acute myeloid leukemia or high-risk myelodysplastic syndrome: predictive prognostic models for outcome. Cancer 106, 1090–1098. 10.1002/cncr.21723 16435386

[B12] Lorgelly P. K. Doble B. Rowen D. Brazier J. Cancer 2015 Investigators (2017). Condition-specific or generic preference-based measures in oncology? A comparison of the EORTC-8D and the EQ-5D-3L. Qual. Life Res. 26, 1163–1176. 10.1007/s11136-016-1443-y 27830513 PMC5376391

[B13] Measurement properties: validity, reliability, and responsiveness (2018). Centers for Disease Control and Prevention. Available at: https://www.cdc.gov/hrqol/measurement.htm (Accessed 10 September 2019).

[B14] Menzin J. Lang K. Earle C. C. Kerney D. Mallick R. (2002). The outcomes and costs of acute myeloid leukemia among the elderly. Arch. Internal Med. 162, 1597–1603. 10.1001/archinte.162.14.1597 12123403

[B15] Mokkink L. B. Terwee C. B. Partick D. L. Alonso J. Stratford P. Knol D. (2010). The COSMIN checklist for assessing the methodological quality of studies on measurement properties of health status measurement instruments: an international Delphi study. Qual. Life Res. 19, 539–549. 10.1007/s11136-010-9606-8 20169472 PMC2852520

[B16] Mukaka M. M. (2012). Statistics corner: a guide to appropriate use of correlation coefficient in medical research. Malawi Med. J. 24, 69–71. 23638278 PMC3576830

[B17] Oliva E. N. Nobile F. Dimitrov B. D. (2013). Development and validation of QOL-E© instrument for the assessment of health-related quality of life in myelodysplastic syndromes. Cent. Eur. J. Med. 8, 835–844. 10.2478/s11536-013-0196-z

[B18] Peipert J. D. Yount S. E. Efficace F. Loefgren C. Pierson R. He J. (2020). Validation of the functional Assessment of Cancer Therapy-Leukemia instrument in patients with acute myeloid leukemia who are not candidates for intensive therapy. Cancer 126 (15), 3542–3551. 10.1002/cncr.32977 32463931

[B19] Schumacher A. Kessler T. Buchner T. Wewers D. van de Loo J. (1998). Quality of life in adult patients with acute myeloid leukemia receiving intensive and prolonged chemotherapy—a longitudinal study. Leukemia 12, 586–592. 10.1038/sj.leu.2400977 9557618

[B20] Shih C.-L. Chen C.-H. Sheu C.-F. Lang H.-C. Hsieh C.-L. (2013). Validating and improving the reliability of the EORTC QLQ-C30 using a multidimensional Rasch model. Value Health 16, 848–854. 10.1016/j.jval.2013.05.004 23947980

[B21] Stalfelt A. M. Wadman B. (1993). Assessing quality of life in leukemia: presentation of an instrument for assessing quality of life in patients with blood malignancies. Qual. Assur. Health Care 5, 201–211. 10.1093/intqhc/5.3.201 8260638

[B22] Watson M. Zittoun R. Hall E. Solbu G. Wheatley K. (1996). A modular questionnaire for the assessment of longterm quality of life in leukaemia patients: the MRC/EORTC QLQ-LEU. Qual. Life Res. 5, 15–19. 10.1007/BF00435964 8901362

