# Supplementary material for: Validated Instruments of Quality of Life (QOL) in Patients With Acute Myeloid Leukemia (AML) and Other Cancers
Source: Front Pharmacol. 2020 Jul 24;11:1109. doi: 10.3389/fphar.2020.01109 (PMC7394002; doi:10.3389/fphar.2020.01109)
Supplement: Supplementary file 1 [file Table_1.docx]

Supplementary Table 1. OVID Search Strategy for Original and Updated Literature Searches.

| **Database** | **Search Strategy** | **Results^1^** | **Results^2^** |
| --- | --- | --- | --- |
| OVID^3^ | [[(Leukemia, Myeloid, Acute/ OR acute myeloid leukemia/ OR ("acute myeloid leukemia" or "acute myelogenous leukemia" or AML).ti,ab OR (acute adj1 myelo* adj1 (leukemia or leukaemia)).ti,ab.) **AND** (("life quality" or QoL or "disease burden*").ti,ab. OR (quality adj1 life).ti,ab. OR (burden* adj1 disease).ti,ab.)] **AND** ((scale* or scor* or measurement* or measur*or questionnaire* or profile or inventory or "NCCN distress thermometer").ti,ab. OR "Surveys and Questionnaires"/ OR (patient health questionnaire/ or Patient Health Questionnaire 15/ or Patient Health Questionnaire 2/ or Patient Health Questionnaire 9/ or questionnaire/ or General Health Questionnaire/ or Patient Health Questionnaire 8/ or Health Assessment Questionnaire/) OR functional assessment/ OR Beck Depression Inventory/ OR (PCM or FACT or FACT-Leu or "NCCN DT" or FACT-G or GACT-BMT or "EORTC QLQ-C30" or QOL-E or HADS or ESAS or POMS or QOL-CS or FACIT-Fatique).ti,ab. OR ("patient care monitor" or "Functional Assessment of Cancer therapy" or "NCCN distress thermometer" or "European organization for research and treatment of cancer quality of life" or "hospital anxiety and depression scale" or "edmonton symptom assessment scale" or "profile of mood state").ti,ab. OR ((functional or assess*) adj3 (profile or scale or questionnaire or scor*)).ti,ab.)] **OR** [[(Leukemia, Myeloid, Acute/ OR acute myeloid leukemia/ OR ("acute myeloid leukemia" or "acute myelogenous leukemia" or AML).ti,ab OR (acute adj1 myelo* adj1 (leukemia or leukaemia)).ti,ab.) **AND** (("life quality" or QoL or "disease burden*").ti,ab. OR (quality adj1 life).ti,ab. OR (burden* adj1 disease).ti,ab.)] **AND** [(validat* adj3 (profil* or instrument or scale or score or measurement or questionnaire or assessment)).ti,ab.]] | 252 | 88 |

^1^Results from original search, run from 1980 through 2018 and limited to English language with duplicates removed; ^2^Results from updated search, run from 2018 to June 2020 and limited to English language with duplicates removed; ^3^Including BIOSIS Previews, Embase, Ovid MEDLINE(R), Ovid MEDLINE(R) Epub Ahead of Print, Ovid MEDLINE(R) In-Process & Other Non-Indexed Citations
